# Cryo-electron tomography of motile cilia and flagella

**DOI:** 10.1186/s13630-014-0012-7

**Published:** 2015-02-02

**Authors:** Takashi Ishikawa

**Affiliations:** Group of Electron Microscopy of Complex Cellular System, Laboratory of Biomolecular Research, Paul Scherrer Institute, OFLG/010, 5232 Villigen PSI, Switzerland

## Abstract

Cryo-electron tomography has been a valuable tool in the analysis of 3D structures of cilia at molecular and cellular levels. It opened a way to reconstruct 3D conformations of proteins in cilia at 3-nm resolution, revealed networks of a number of component proteins in cilia, and has even allowed the study of component dynamics. In particular, we have identified the locations and conformations of all the regular inner and outer dyneins, as well as various regulators such as radial spokes. Since the mid 2000s, cryo-electron tomography has provided us with new knowledge, concepts, and questions in the area of cilia research. Now, after nearly 10 years of application of this technique, we are turning a corner and are at the stage to discuss the next steps. We expect further development of this technique for specimen preparation, data acquisition, and analysis. While combining this tool with other methodologies has already made cryo-electron tomography more biologically significant, we need to continue this cooperation using recently developed biotechnology and cell biology approaches.

In this review, we will provide an up-to-date overview of the biological insights obtained by cryo-electron tomography and will discuss future possibilities of this technique in the context of cilia research.

**Electronic supplementary material:**

The online version of this article (doi:10.1186/s13630-014-0012-7) contains supplementary material, which is available to authorized users.

## Review

### Why electron tomography?

3D structural analysis from transmission electron microscopy, especially cryo-EM, has been playing indispensable role in motor protein research as a potential method to analyze 3D structure of complexes of motor and cytoskeletal proteins. The small sizes of myosin and kinesin heads allow these motors to fully decorate filaments at stoichiometric ratios (one myosin to one actin, one kinesin to one beta-tubulin). Electron micrographs of fully decorated actin and microtubule filaments, which are helical, provide an image of motor proteins with full coverage of view angles and thus allow 3D reconstruction at pseudo atomic resolution of myosin/actin [1,2] and kinesin/microtubule [3,4]. Since muscle contraction and intracellular transport are linear motions, *in vitro* reconstituted filaments decorated by motors can reasonably be considered as simplified systems of *in vivo* motility. This approach is applied successfully to unveil the regulatory mechanism of muscle contraction by calcium ions as well [5,6]. In dynein research, however, the extraordinarily large size (approximately 4,500 amino acids) of this motor protein prohibits full decoration of the microtubule. For microtubules sparsely decorated by whole dynein heads, single particle analysis can be applied. This method merges micrographs of dyneins on the microtubule under the assumption that they share an identical 3D structure at random orientations. In spite of limited resolution (approximately 20 Å) due to flexibility of this still gigantic protein, dynein on the microtubule has been visualized [7,8]. Full decoration by dynein stalks is possible, which has enabled visualization of microtubule binding of dynein at pre- and post-power stroke states at pseudo atomic resolution [9,10]. Single particle analysis of dynein heads without microtubules enabled the conformational change induced by nucleotides to be visualized [11,12].

To investigate structural mechanisms of more complex phenomena such as ciliary bending motion, higher order structure must be investigated. Since no *in vitro* reconstituted system reproduces ciliary bending, *in vivo* imaging is the most promising approach to describe structural bases of ciliary function. *In vivo* electron microscopy must take a different approach from *in vitro*, since no two objects share an identical 3D structure. We must record projections of one object from various angles and merge them into the 3D structure. This method is called electron tomography. Fortunately, recent development of electron tomography, which was enabled by technical developments such as the stable specimen stage, high sensitivity detection, alignment algorithm, and optics for high contrast, took place synchronously with the demand of 3D structural analysis of cilia.

**Figure 1:**
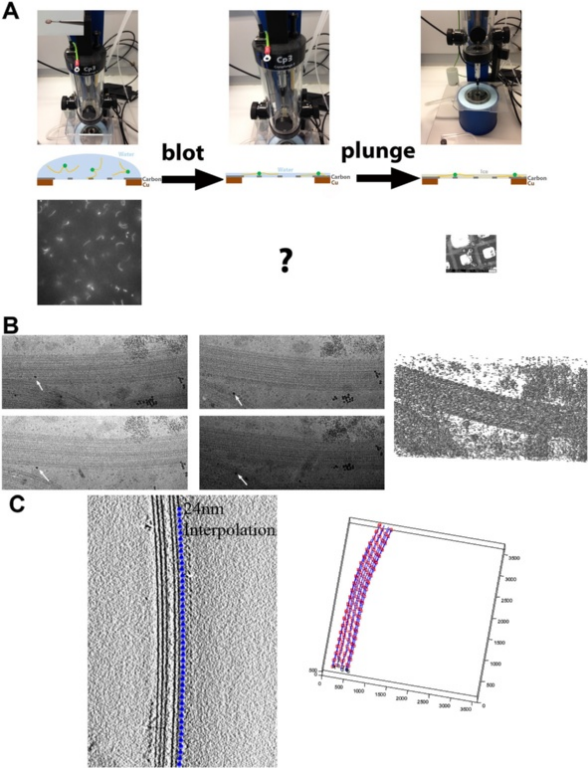
**Process of cryo-electron tomography. (A)** Plunge freezing for cryo-electron tomography and microscopy. Left: before blotting (EM grid with mounted specimen solution is shown in the inset of the top panel). Center: after blotting. Right: after plunging. Upper panels: freezing apparatus (Gatan Cp3). Middle panels: schematic diagrams to describe the side view of the grid and the specimen. Grey: holey carbon membrane. Brown: cupper mesh. Bottom panels: *Chlamydomonas* flagella and cells before blotting and after plunging. The specimen condition after blotting cannot be observed with the current instruments. **(B)** Electron micrographs and a tomogram. A fiducial gold marker is shown by arrows. **(C)** Specific image analysis strategy of subtomogram averaging in our research on cilia, based on periodicity.

At the same time, cilia research has been one of the most typical targets of electron tomography and is a prototype for its methodology due to the advantage of the characteristic “9 + 2” structure [13]. As shown in Figure 1B, the low tomogram obtained from ice-embedded (cryo) samples has an extremely high noise level. To extract conformational information of molecules, we must box out subvolumes of target molecules from the entire tomogram and average them. Before averaging, each subvolume must be reoriented to have the same direction. This process is called subtomogram alignment and averaging. Normally, extracting subtomograms from noisy tomograms and aligning them is not straightforward. However, in motile cilia, we can locate the approximate position of target molecules such as microtubule doublets, axonemal dyneins, and radial spokes based on the ninefold symmetry and 96-nm periodicity along each doublet (please note that this periodicity and symmetry have exceptions in *Chlamydomonas* flagella; see Asymmetrical arrangement of inner arm dyneins and other proteins in *Chlamydomonas* flagella). This structural property of cilia eased subtomogram extraction, alignment, and averaging and allowed electron tomography of cilia to further the application of this technique in various biological systems [14].

### History of electron tomography of cilia

Computational imaging of cilia based on electron microscopy has long history. In fact, the image averaging technique using 96-nm periodicity was applied to electron micrographs of resin-embedded, stained, and sectioned cilia before electron tomography and unveiled the arrangement of some dynein heavy, light, and intermediate chains [15,16].

Cryo-electron tomography of cilia was pioneered in 2002 [17]. However, the first 3D structure analyzed by electron tomography and subtomogram averaging was published by Lupetti’s group using freeze-fracture deep-etched sperm axoneme from the cecidomid dipteran *Monarthropalpus flavus* used. They utilized an unusual planar axoneme surface with many microtubule doublets with outer arm dyneins forming 2D arrays [18]. The averaged structure of the replica presents the molecular surface of dyneins which is nearly identical to that from cryo-EM tomography made based on ninefold symmetry of the axoneme [19-21]. Since then, cryo-electron tomography and subtomogram averaging have been successfully revealing structures of the axoneme. Recently, 3D structural studies have expanded to ciliary/flagellar structures out of axonemal periodicity. Intraflagellar transport (IFT), paraflagella, and the basal body are targets of this technique, which we will review in sections IFT and other structures and Basal body.

### Cryo-electron tomography of cilia

In this article, I will mainly review works on cilia structure using cryo-electron tomography. One advantage of cryo-preparation is preservation of native structure at the molecular level, while other preparation methods such as chemical fixation and staining, high pressure freezing followed by freeze substitution, and freeze-fracture deep-etch suffer from artifacts caused by fixation and staining. The most serious disadvantage of cryo-tomography is low contrast and poor signal-to-noise ratio (S/N). Cryo-electron tomography provides enormous opportunity when combined with subtomogram averaging, whereas for simple morphological studies such as direct observation of cytoskeletal networks, other preparations at room temperature might be more suitable. Indeed, unique knowledge about the arrangement of microtubule doublets and singlet microtubules was obtained by high pressure frozen and freeze-substituted cilia [22]. The outstanding advantage of cryo-electron tomography appears when identical molecules can be detected, aligned, and averaged. Therefore, 96-nm periodicity and (pseudo) ninefold symmetry of the axoneme is a benefit for *in vivo* structural analysis by cryo-electron tomography.

## Methodology of cryo-electron tomography for cilia

### Freezing

There are two methods to embed biological specimens in amorphous ice (vitrification): plunge freezing and high pressure freezing. Plunge freezing (Figure 1A), used by most of the works mentioned in this review (including all of our publications), is a simpler method to freeze specimens in a thin (<0.5 μm) water layer by plunging it into cryogen (liquid ethane or liquid propane). A 3–5 μl drop of specimen (either isolated cilia or ciliated cells in buffer) is mounted on a holey carbon grid. To make a thin solution layer, excess liquid on the grid is blotted with filter paper (self-made or commercially available instruments from FEI, Gatan, or Leica can be used). The blotted grid is plunged into cryogen at liquid nitrogen temperature. Freezing occurs within microseconds.

Due to the diameter of cilia (250–300 nm), plunge freezing is suitable for cilia isolated from the cell body. When cilia as appendages of cells are plunge-frozen, it is difficult to observe the proximal part due to thick ice made by the cell body, while the central and the distal part are embedded in thin ice and provide enough contrast for cryo-ET. To observe the proximal region and the basal body in the cell, plunge-frozen cells must be sectioned. For even thicker cells and tissues, high pressure freezing is necessary. In this approach, bulky specimen is frozen under approximately 2,000 bar pressure and sectioned by cryo-ultramicrotome. With this method, thicker specimens than the axoneme, such as intact flagella from *Trypanosoma brucei* [23] or primary cilia from *Caenorhabditis elegans* [24], were visualized by cryo-electron tomography.

### Instrumentation for cryo-electron tomography

To be capable for cryo-tomography, the transmission electron microscope must be equipped with special parts. To obtain high contrast from ice-embedded specimen, a field emission gun (FEG) is essential. A stable specimen stage with computer control, high precision, and high tilt (at least 60°) is necessary. Tomographic data acquisition is normally a long, sustained process (acquisition of one tomogram takes 0.5–1 h, and tens of datasets are necessary for averaging). For long, continuous acquisition, a specially designed cryo-polepiece to reduce ice contamination is helpful. For data collection from flagella/cilia, at whatever acceleration voltage, an energy filter is requisite because of the thickness of the specimen. Micrographs must be recorded with digital detectors, which have higher sensitivity than photographic films. We are using standard digital detectors—CMOS and CCD cameras with scintillators. The recent progress of direct electron detectors, which brought a breakthrough in single particle analysis and enabled atomic resolution of *in vitro* structural analysis [25], may allow us to collect data with lower electron doses and therefore at higher resolution, although there is no systematic comparison yet reported.

In tomographic data acquisition, the stage can be tilted only up to certain tilt angle, 60°–80° depending on the instrumentation. This limitation causes partial loss of structural information called missing wedge and results in an artifact in the tomogram [26]. When double-axis tilt is possible, the missing information is in a pyramid shape (missing pyramid). The missing information can be compensated by averaging subtomograms oriented differently. During axoneme analysis, tomographic data collection of axonemes oriented parallel to the tilt axis is recommended because of its advantage of nine microtubule doublets arranged with 40° difference of missing wedges around the axis, which enables complete coverage of all the view angles without any missing wedge. Higher electron dose improves S/N but causes more radiation damage. The dose for our data collection varies from 30e^−^/Å^2^, when we pursue molecular structure at the highest resolution, to 60e^−^/Å^2^ (accelerating voltage is 200 kV), when we only need to locate target molecules [27].

## Analysis

3D image analysis of cilia from cryo-electron tomography consists of two parts: tomogram reconstruction and subtomogram averaging. The first part is common among various projects using electron tomography (Figure 1A,B) and can be done with conventional tomography reconstruction packages [28,29]. For cryo-tomography, we use 10- to 15-nm gold particles as fiducial markers (arrows in Figure 1B). Reconstruction is computed by R-weighted backprojection.

The second part of cilia analysis is subtomogram averaging (Figure 1C). In the axoneme analysis, cubic volumes, enough to cover 96-nm periodic units, are extracted from tomograms computationally (called subtomograms). They are aligned to have the same orientation based on cross correlation and averaged (a detailed alignment procedure is described elsewhere [13]). This process improves S/N, compensates missing wedges, and provides 3D structure under the assumption that all the subtomograms involved in the average share an identical structure. This assumption is not trivial—under high noise level and with missing wedge, it is hard to judge if subtomograms have the same structure or not. Heterogeneity in averaging may result in missing density, degraded resolution, or artifacts. To deal with structure with heterogeneity which is not straightforwardly detectable, for example dynein structure in the presence of nucleotides, we need a process called image classification of subtomograms. Statistical analysis is used to classify subtomograms into subgroups and average them separately (subaverages). We developed an image classification algorithm using cross-correlation-based template matching [30]. For unsupervised classification without bias from the templates, we carried out multivariate statistical analysis with missing wedge filled with averaged images [30].

## Ciliary structure revealed by cryo-electron tomography—I. Dynein

**Figure 2:**
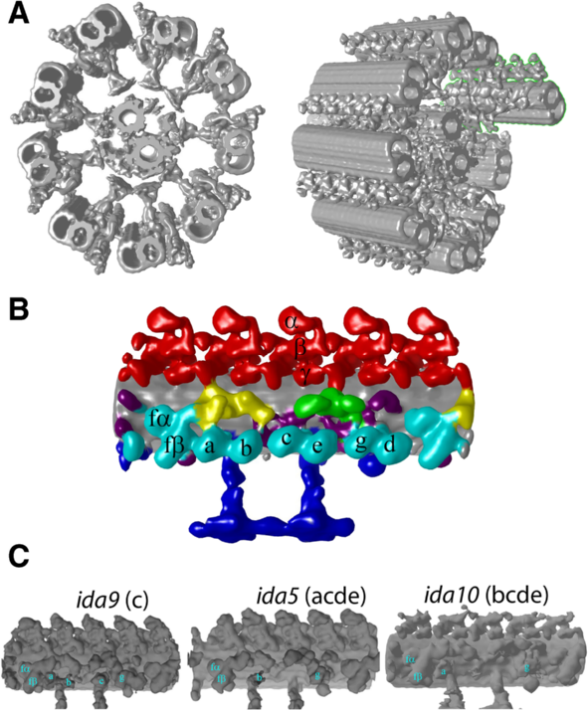
**3D structure of** ***Chlamydomonas*** **flagella reconstructed by cryo-electron tomography and subtomogram averaging. (A)** Structure of the entire axoneme by fitting averaged 96-nm periodic units to a tomogram. One MTD, discussed in the following sections, is enclosed by green lines in the right panel. **(B)** Averaged 96-nm unit. Red: outer dyneins. Cyan: inner dyneins. Blue: Radial spokes. Yellow: IC/LC of dynein f. Green: DRC. Grey: microtubule doublets (MTDs). Purple: unidentified density. Dynein isoforms were assigned based on **(C)**. **(C)** Flagella structure of *Chlamydomonas* mutants used for identification of dynein isoforms. Missing IDA species are indicated.

In Dynein arrangement and localization in the axoneme to Nexin/DRC and other interdoublet linkers, MIPs, we will review 3D structure of the 96-nm periodic unit from *Chlamydomonas* flagella, *Tetrahymena* cilia, sea urchin sperm flagella, and mouse respiratory cilia, as averaged along a microtubule doublet (MTD) and among nine MTDs (Figure 2A,B). Based on these structures, we will discuss the arrangement and conformation of dyneins, radial spoke proteins, and other molecules. In this section, we focus on dynein. There are a number of questions regarding dynein in cilia at multiple scales, from the scale of single dynein molecule to the organelle level. We will review these questions and our effort to answer them using cryo-electron tomography, from the smaller scale to the larger.

### Dynein arrangement and localization in the axoneme

Axonemal dyneins are a main driving force of ciliary bending motion. Unlike cytoplasmic dynein, there are a number of isoforms in axonemal dyneins. Therefore, one of our initial research focuses was to locate these isoforms in the axoneme. In *Chlamydomonas*, 16 genes of dyneins are reported, among which one is cytoplasmic dynein, three are outer arm dyneins, and 12 should be inner arm dyneins [31,32]. Eight of the inner arm dyneins were detected biochemically [16], and the other four, called minor dyneins, were located at the proximal region of the flagella [33] (detail in Asymmetrical arrangement of inner arm dyneins and other proteins in *Chlamydomonas* flagella). Within reconstruction of one 96-nm periodic unit of one of nine MTDs (Figure 2A), there are eight inner arm dynein heads (cyan in Figure 2B and the Additional file 1: Video) and four rows of three stacking outer arm dynein heads (red in Figure 2B) found as ring-shaped approximately 12-nm objects. We identified inner dynein isoforms, comparing structures from various mutants lacking dyneins [34,35]. As an example, in Figure 2C, structures of mutants which lack dynein c, dyneins a/c/d/e, and dynein b/c/d/e, respectively, are shown. These mutants were used to identify dynein isoforms in the tomogram. Six single-headed dyneins (a, b, c, e, g, d) form three dyads. Each dyad is connected to one radial spoke (RS) [34]. Interestingly, according to biochemical works [36], each dyad should contain actin and p28 or centrin.

Identification of light chains (LC) and intermediate chains (IC) started recently. After description of the overall structure of IC/LC complex of dynein f [35], the locations of IC138 and modifier of inner arms (MIA) complex were determined [37,38]. MIA is at an interface of IC/LC and DRC by structural analysis of deletion mutants [38]. Biotin carboxyl carrier protein (BCCP) tagging enabled location of IC2 between ODA and IC/LC [39].

### Asymmetrical arrangement of inner arm dyneins and other proteins in *Chlamydomonas* flagella

**Figure 3:**
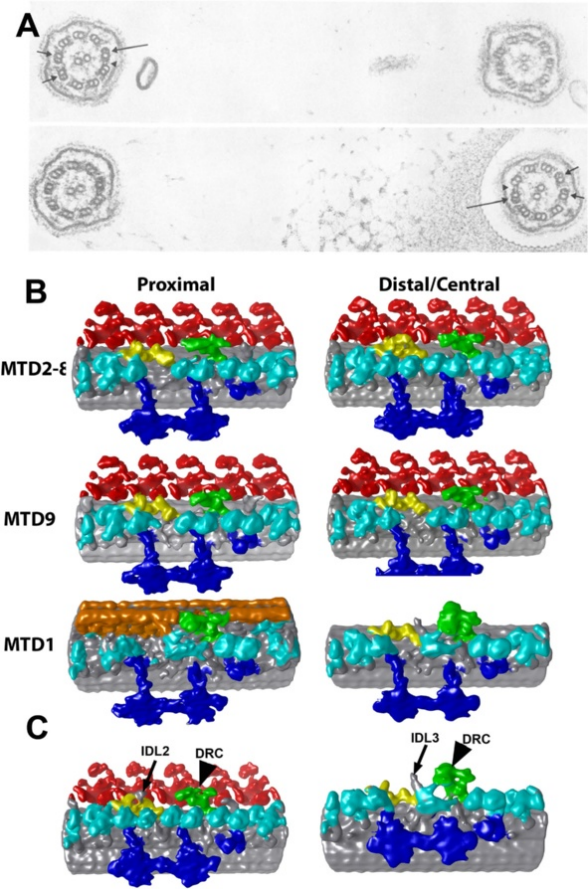
**Asymmetry of molecular arrangement in** ***Chlamydomonas*** **flagella. (A)** Electron micrographs of cross sections of flagella in *Chlamydomonas* cells (without deflagellation), modified from [40]. MTD1 and MTDs 5/6 are indicated by a long and short arrows. The 1–2 bridge is shown by arrowheads. **(B)** Structure of nine MTDs from the proximal and the distal regions, averaged separately. Red: ODA. Cyan: IDA. Dark blue: RS. Yellow: IC/LC. Green: nexin/DRC. Orange: 1–2 bridge. Modified from [35]. These structures are available in EM Databank (EMD2113-2130). **(C)** 3D structure of MTD1 (right) and MTD4 (left). From EMD2113 and EMD2119. The arrows indicate two extra linkers (IDL2 and IDL3). The arrowheads indicate nexin/DRC.

Asymmetry of outer dynein arm (ODA) in *Chlamydomonas* flagella was known based on electron microscopy of plastic-embedded cells [40] (Figure 3A). Since their study utilized microscopy of the intact cells, they could identify nine microtubule doublets (MTDs) with respect to the other flagellum, in which the apposed MTD was numbered MTD1. MTD1 was proved to lack ODAs. They also reported dense linkers specifically connecting the proximal region (within 1–2 μm from the basal body) of MTD1 and MTD2 (1–2 bridge). These linkers are arrayed with 8-nm spacing [35].

We applied cryo-ET to explore further detail of the asymmetry, both circumferentially and longitudinally [35,41]. We averaged subtomograms extracted from individual MTDs and averaged separately. MTDs can be identified based on the fact that MTD1 lacks ODA. The proximal and the distal ends are identified in the micrograph as explained elsewhere [13]. The proximal region is defined as an area from approximately 2 μm from the proximal end and subtomograms from that region were averaged separately. We call the rest of the area, which covers more than two thirds of the full-length flagella, the distal region. We did not find heterogeneity of the dynein arrangement inside the distal region. In total, we obtained 18 averages (density maps are available in EM Databank as EMD2113-2130).

Presence of dynein isoforms on all the MTDs in the proximal and the distal regions is shown in Figure 3B. While MTDs 2–8 in the distal region are fully decorated by eight inner dyneins (dyneins a, b, c, d, e, f—which is a dimer, and g) (top right of Figure 3B), other parts lack one or more inner dynein isoforms [35,41]. The locus of dynein b is empty on MTD1 and MTD9 (middle and bottom right of Figure 3B). Dynein b is missing also on all the MTDs in the proximal region (left panels of Figure 3B). Furthermore, molecular conformation at the loci of the two inner arm dyneins c and e is unusual in MTD1—the height of the heads at this position is lower than those of other inner dyneins, suggesting either irregular folding of dyneins c and e or replacement by other molecules (bottom right of Figure 3B). MTD1 also lacks dynein f at the proximal region. These results indicate that delivery and assembly of inner arm dyneins are not uniform all over the axoneme, the mechanism of which we do not know. There might be a designed specific binding of dynein isoforms on MTDs or a targeted delivery system.

In addition to the absence of dyneins, there are cases of replacement of some dynein isoforms with others, which takes place in the proximal region. One example of our strategy to understand this replacement, even at resolutions which are not high enough to distinguish isoforms, is as follows. A *Chlamydomonas* mutant *ida10* lacks inner arm dyneins b, c, d, and e, as well as minor dyneins DHC3 and DHC4. In structure, the subtomogram averaging shows an empty locus at the position of dynein d, as expected. However, when we averaged subtomograms which were extracted from the proximal region only, this locus had a density of dynein. Since this density cannot be dynein d, it must be assigned to something else. DHC11, the only minor dynein contained in this strain, is the most likely candidate. This hypothesis is consistent with immunofluorescence localization, showing this minor dynein localized at the proximal region [33]. This led us to the conclusion that minor dyneins replace major dyneins in the proximal region [35].

Averaging of subtomograms from separate regions and MTDs gave us further insight into the circumferential and longitudinal asymmetry of the axonemal structure in *Chlamydomonas* flagella. In addition to nexin/DRC, which protrudes between inner dynein arm (IDA) (between dyneins e and g) and ODA and links all the adjacent MTDs, we found two linkers, which we named IDL2 and IDL3 (Figure 3C). All these linkers extend from one MTD to the next, between IDA and ODA. IDL2 extends only from MTDs 4, 5, and 9, located between dyneins a and b, associated with intermediate and light chains (IC/LC) of dynein f. IDL3 links MTD1 and MTD2 and protrudes from the interface of IC/LC and DRC. We found further asymmetry in the axoneme; there are more connections between IC/LC and dyneins and between nexin/DRC and ODA in some pairs of adjacent MTDs. Although we do not have space here to go into detail, they are described in [35].

We do not have direct evidence of how the asymmetry found in this study plays a role in flagellar bending and waveform determination. However, the coincidence between the orientation of the asymmetry and the direction of bending inspired us to build a following model. All the extra linkers exist on or near the bending plane, which involves MTD1 and between MTDs 5 and 6. These linkers make the bending motion planar, by restricting sliding between MTD 9 and 1, 1 and 2, 4 and 5, and 5 and 6. Lack of dynein b on MTDs 1 and 9 limits the sliding force at one side of the axoneme, enabling asymmetric waveforms. This model should be tested by comparing axonemes with different waveforms. One question that is yet unanswered is how *Chlamydomonas* flagella change their waveform to be symmetric in the presence of high calcium concentration.

Further study should be done to investigate different species as well as mechanisms of asymmetry generation, that is, if asymmetry derives from basal bodies or is generated during the growth of the axoneme. Detailed investigation of IFT cargos may give us insight into mechanisms of asymmetry.

### Structure of dynein heavy chains

**Figure 4:**
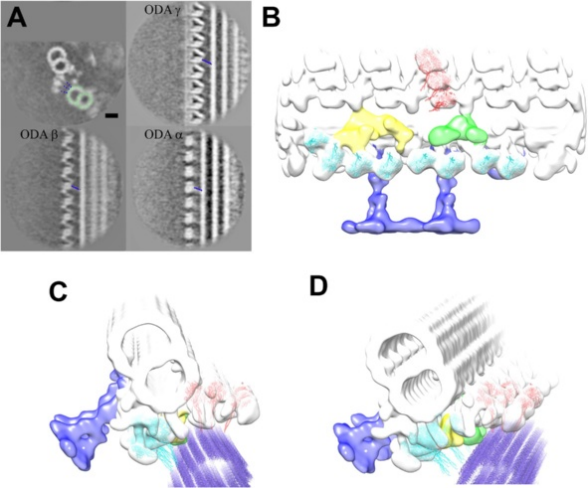
**Atomic model fitting to the 3D structure averaged from the tomograms of** ***Chlamydomonas*** **flagella. (A)** Sections of our tomographic reconstruction. Protofilaments and dynein stalks are indicated in green and blue, respectively. Modified from [41] **(B-D).** Fitting atomic models of cytoplasmic dynein heads (3VKG; the microtubule binding domain is not shown) [42] to our tomography [35] (EMD2117). Red: ODA. Cyan: IDA. Blue: MTD. **(B)** View seen from the adjacent MTD. Left: proximal end. Right: distal end. **(C, D)** Views from the proximal end.

**Figure 5:**
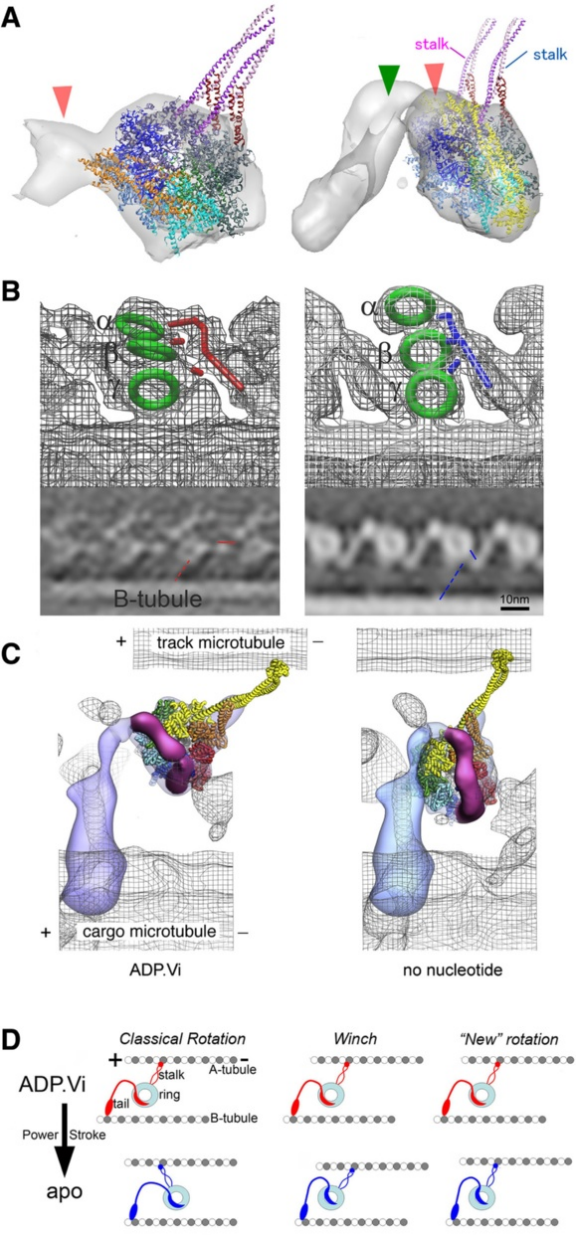
**Structural change of dynein induced by nucleotides. (A-C)** Left: structure with ADP.Vi (pre-power stroke). Right: structure without additional nucleotide (apo; post-power stroke). **(A)** Tomography structure of mouse respiratory cilia ODA, consisting of two dyneins, which highlights relocation of the linker with respect to the AAA ring. The linker is shown in orange and yellow in the ADP.Vi and apo forms, respectively [45]. **(B)** Tomography structure of *Chlamydomonas* ODA, showing shift of the head (green rings) and orientations of the stalk (blue and red dotted lines) as well as the neck domains and N-terminal tails (red and blue solid lines). From [30] with permission. **(C)** High resolution single particle structures by the Burgess group fitted to our tomogram. This fitting suggests rotation of the stalk. From [12] with permission. **(D)** Schematic diagrams of three hypotheses of the dynein power stroke.

Here, we will overview the conformation of dynein heavy chains mainly from *Chlamydomonas*, in the absence of additional nucleotides. The stalks of outer arm dyneins were found to connect the rings and the adjacent microtubule (Figure 4A). The tails of both inner and outer dyneins extend from the rings toward the distal end (called the neck region; red and blue lines in Figure 5B). These features enabled us to fit atomic models of dynein motor domains from cytoplasmic dynein [42,43] (Figure 4B-D), without modification of atomic structure at this resolution (approximately 30 Å). The fitting to the outer arm dyneins is highly reliable based on the stalks, while fitting inner dyneins is based on cross correlation and is limited by resolution.

The orientation of the ring and the stalk are similar (the stalk tilts toward the proximal end) to each other in all the three outer arm dyneins [41] (blue dotted lines in Figure 4A). The orientation of inner dynein stalks is also likely tilted toward the proximal end, judging from the fitting of the atomic models to our tomography structure (blue atomic models in Figure 4B-D and in the Additional file 1: Video). This suggests that inner and outer dyneins are arranged, in principle, to cooperate force generation in the same (or similar) direction. Upon close inspection, we found variety of the head orientation between dynein heads (Figure 4B; Additional file 1: Video).

In outer dynein arms from mouse respiratory cilia (Figure 5A) and *Chlamydomonas* (Figure 5B), alpha and beta dynein heads are connected, suggesting interaction. The interface between the gamma dynein head and the microtubule is occupied by two separate densities. One at the external site is likely the docking complex, judging from the position on the microtubule [44] (Additional file 1: Video). While the tails can be traced straightforwardly in inner arm dyneins, the conformation of outer arm dynein tails appears more complex and is open to interpretation.

### Dynein structural change induced by nucleotides

In addition to the atomic structure of cytoplasmic dynein in the post-power stroke state produced by X-ray crystallography (ADP: [42]; Apo: [43]), nucleotide-induced structural change of axonemal and cytoplasmic dyneins were analyzed by single particle cryo-EM [12] at approximately 20-Å resolution. According to their 3D reconstruction, upon addition of ATP and vanadate, which makes dynein bound by ADP and vanadate (ADP.Vi), the linker domain moves from AAA5 (post-power stroke) to AAA2 (pre-power stroke) (Figure 5C). Our group [45] and the Nicastro group [46] fit atomic models of the linker and the ring separately and reached the same conclusion (Figure 5A). This relocation of the linker on the AAA ring implies a force generation mechanism of dynein. However, to reveal the mechanism of dynein motility on the microtubule, analysis of dynein with microtubules is needed. Here, we discuss the mechanism of force generation by fitting of tomography, single particle analysis, and atomic structure. In tomography, the angle of the stalk with respect to the microtubule can be measured directly, while discussion at the atomic level is enabled by fitting high resolution atomic models or single particle structures.

There were two hypotheses proposed as models of the dynein power stroke: the rotation hypothesis and the winch hypothesis. In the rotation hypothesis, the ring and the stalk rotate together to push the adjacent microtubule toward the plus end, while in the winch hypothesis, the head shifts and pulls the adjacent microtubule toward the plus end (Figure 5D). In previous work, we stated that our analysis supported the winch hypothesis based on the fact that the position of dynein heads shift approximately 8 nm toward the distal end (microtubule plus end) upon addition of ADP.Vi, which clearly supports “winch” (Figure 5B) [30]. Moreover, the absence of a large change in orientation of the stalk (as shown in Figure 5D left) seemed to exclude the possibility of the rotation hypothesis, as previously proposed (“classical rotation hypothesis”). However, further scrutiny may indicate that one cannot choose “rotation” or “winch” exclusively. A careful look indicates a slight counterclockwise (5–20°) rotation of the stalk. In the pre-power stroke structure of the single particle analysis fitted to our tomography structure, the angle of the stalk with respect to the microtubule is approximately 40°, whereas in the post-power stroke it is approximately 60° (Figure 5C) [12]. The rotation seen in tomography from our group and Nicastro’s group is not so much, but still approximately 5° rotation is observed in the same direction as seen in single particle analysis; the angle of the stalk with respect to the microtubule is approximately 50° and approximately 55° in the pre- and post-power stroke forms, respectively (Figure 5B) [30]. This motion cannot be explained as a passive result of dynein shift—if it were, the rotation should be in the opposite direction. These results suggest that there should be a driving force to rotate the stalk toward the flagellar tip. Although we do not know if this rotation is a driving force of the dynein power stroke, this might suggest another type of the rotation hypothesis—a tiny rotation of the dynein stalk is linked to the power stroke in combination with a shift of dynein heads induced by reconformation of the linker.

### Moving step of dynein dimers

**Figure 6:**
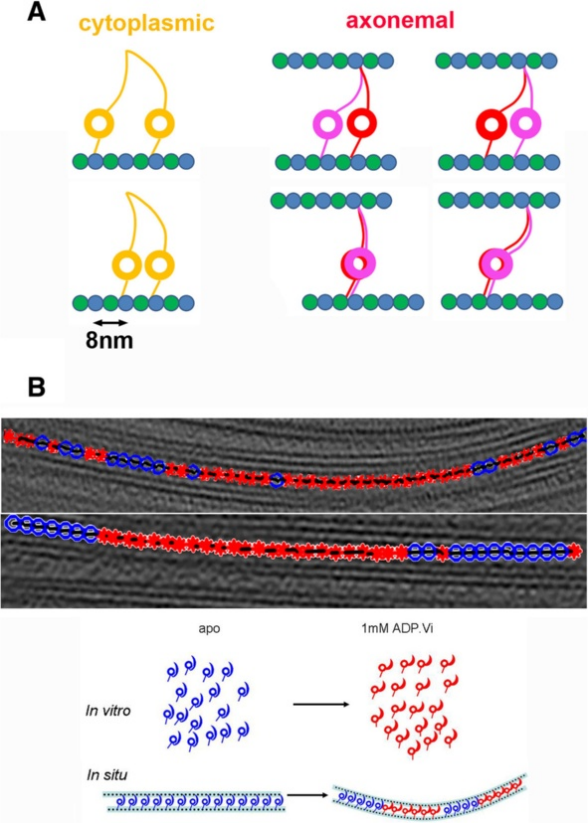
**Behavior of multiple dyneins in the axoneme. (A)** Schematic diagram of the motion of cytoplasmic dyneins revealed by *in vitro* motility assays of yeast dynein and axonemal dynein dimers from mouse respiratory cilia *in vivo* analyzed by cryo-electron tomography. Left: cytoplasmic dynein (homodimer). Two heads can be 16 nm or longer apart [47,48]. They are rarely at the same position. Center and right: axonemal dynein. The distance between the two heads are either 0 nm or 8 nm [45]. **(B)** Distribution of heterogeneous structures of ODAs forming an array on MTD in the presence of ADP.Vi, revealed by cryo-electron tomography and image classification of *Chlamydomonas* flagella [30]. Upper panels: image classification of ODAs in the tomogram. Red: ODA in the ADP.Vi form. Blue: ODA in the apo form. Middle panels: schematic diagram of isolated dyneins in the presence and absence of ADP.Vi. With 1-mM ADP.Vi, the ADP.Vi form dominates. Bottom panels: ODA in flagella. Even in the presence of ADP.Vi, many ODAs remain in the apo form. Interestingly, two conformations form cluster as seen in the top panels.

The behavior of dynein dimers in the axoneme is a question that has yet to be resolved. For cytoplasmic dynein, *in vitro* motility assay studies with two heads of the same monomer labeled with different fluorescent dyes gave insight into the steps of dynein motility. It was reported that during the microtubule-based motion of dynein dimers in the presence of ATP, two heads step alternatingly, either one head passing the other (hand-over-hand) or the trailing head catching up with the other (inchworm). According to two recent studies of yeast dynein by *in vitro* motility assay, two heads are rarely (<20%) at the same position and the average distance between the two heads is 18 nm [47,48]. Axonemal dynein (dynein f in *Chlamydomonas*) forms heterodimers in IDA. Outer dynein arms of many species (vertebrate, echinoderm, arthropod, Mollusca, Platyhelminthes, fungi, etc.) also form heterodimers. We tried to understand if axonemal dynein dimers behave similarly to yeast dynein or not [45]. Although cryo-EM can observe only snapshots and not dynamic motion, we can discuss the difference between *in vitro* motility assays and structural analysis, by comparing the statistics of the distance between the two heads (Figure 6A). In the tomogram of mouse respiratory cilia in the presence of 1-mM ATP, the distance between the two heads is either 8 nm or 0 nm (right panels of Figure 6A). The two heads are either in the same position (both in the pre-power stroke or both in the post-power stroke) or 8 nm apart from each other (one dynein in the pre-power stroke and the other in the post-power stroke). This is unlike the 18-nm distance of cytoplasmic dynein dimers. The probability to have two heads in the same position is 50%, highlighting the difference from the *in vitro* motility assay of cytoplasmic dynein [45].

A few interpretations could explain the discrepancy between the *in vitro* motility assay of yeast cytoplasmic dynein and cryo-electron tomography of the axonemal dynein. Motility of axonemal dyneins might be of shorter steps than cytoplasmic dynein, allowing for only 8-nm distance between the two heads, while cytoplasmic dynein allows 18 nm. The distance between two axonemal dynein heads can be limited due to the spatial constraint on MTDs. *In vitro* motility assays of separately labeled axonemal dynein dimers could examine this hypothesis. The discrepancy might also be due to the fact that yeast dynein lacks the C-terminal domain. When two heads of axonemal dynein stack on top of the other, the C-terminal domain is located at the interface (Figure 5A). Removal of the C-terminal domain may reduce affinity between the two heads and separate them further apart. This could be proven with *in vitro* motility assays of cytoplasmic dynein from other species.

### Structural heterogeneity of dynein and the bending mechanism

While individual dynein motors make sliding motion on the microtubule, they generate bending when integrated into an axoneme. Therefore, a description of a group of dyneins in the axoneme is a key to understanding the bending mechanism. Judging from the intact waveform of *oda1* (lacking entire ODA), IDA is considered to be the main component to determine the waveform [49]. Mutants lacking RS or CP are paralyzed under physiological conditions, leading us to the model of IDA regulation by RS/CP. However, it is known that bending motion of mutants lacking RS or CP is recovered under special nucleotide condition (low ATP, excess of ADP, etc.) in the presence of ODA [50], suggesting there can be another pathway to generate bending motion by ODA, independent of RS/CP (reviewed in [51]). Our 3D image classification implies distribution of ODA in different conformations along MTDs (Figure 6B). This pattern varies among nine MTDs, although we could not find a rule to correlate the pattern of dynein structural heterogeneity on nine MTDs. We analyzed ODA structures from *Chlamydomonas* flagella under various nucleotide conditions. ODA structures are classified into two categories: the apo conformation and the ADP.Vi conformation (blue and red dots in Figure 6B). The ADP.Vi and apo conformations correspond to the pre- and post-power stroke states, respectively. Nearly all the ODAs have the apo conformation in the presence of apyrase (an enzyme which hydrolyzes ATP and ADP to AMP). Flagella without any addition of nucleotide or apyrase are dominated by ODA in the apo conformation but contain a few ODAs in the ADP.Vi conformation, probably due to nucleotides leftover from the cells. With the addition of ATP, structure is heterogeneous, which is reasonable, since ATP will be hydrolyzed by dynein during the EM grid preparation and some of the ODAs are in the post-power stroke state.

Interestingly, even with the addition of ATP and vanadate, heterogeneity of the structure was observed. In the presence of ATP and vanadate, dynein should be fixed in the ADP.Pi state (pre-power stroke), after hydrolyzing one ATP and releasing phosphate (Pi)—vanadate (Vi) is trapped into a pocket, which supposed to be occupied by phosphate (Pi), and inhibits the further ATP hydrolysis cycle [52]. Upon addition of ADP.Vi, isolated dyneins turn their structure to the post-power stroke configuration [11] (middle panel of Figure 6B). On the contrary, behavior of dyneins in flagella is quite different from isolated dynein, in that dynein conformation shows heterogeneity. While half of ODAs in flagella in the presence of ADP.Vi are in the ADP.Vi conformation, the rest of the ODAs stay in the apo conformation (top of Figure 6B). Moreover, these two conformations do not appear randomly but apparently show patterns—on some MTDs, they form clusters. On some MTDs, consecutive 10–20 ODAs are in the ADP.Vi form, while the next row of ODAs are in the apo form, even in the presence of ADP.Vi [30] (top of Figure 6B). This indicates that the ATPase (and the power stroke) cycle of dynein in the axoneme works differently from that of free dynein *in vitro*. To examine this further, we carried out structural and functional studies of MTDs split from the axoneme. Activity of dynein ATPase of split MTDs is 2.2 times enhanced upon addition of microtubules, as expected (microtubule-activated dynein ATPase) [53]. However, in the axoneme, which should correspond to the microtubule-activated ATPase, dynein ATPase was suppressed to 0.4 times [54]. This implies an unknown mechanism to suppressively control outer and inner dynein ATPase in the axoneme.

One possible interpretation of this phenomenon is that, upon nucleotide binding to dynein, there is a cooperative influence on the adjacent dynein, either positive (within the cluster) or negative (between the clusters). Another interpretation is the presence of a mechano-sensing function of dynein—when the axoneme is bent, dynein senses a strain and changes gears to another state. These two working hypotheses are not necessarily mutually exclusive. Our structural analysis demonstrated heterogeneity of ODA conformations along split MTD, indicating neither the whole axoneme nor the bending force is necessary to cause structural heterogeneity [54]. However, in split MTDs, we did not find cluster formation, which may indicate a role of an external force needed to switch gears of dynein. Recently, the Nicastro group showed that the ODA conformation on the outer MTDs of the sea urchin sperm flagella are dominated by the pre-power stroke conformation (the same as our ADP.Vi form), while those on the inner MTDs are in the apo conformation [46]. Their analysis was done using sperm flagella, which, probably before being blotted by filter paper made bending motions on EM grids. The ODA conformation reflects either active motion (in the case that bending motion continues after blotting), in which MTDs at both sides of the axoneme switch between active and inactive states as proposed based on ultrastructure of gill cilia and their splitting patterns [55,56] or conformations caused by strains in the bent axoneme (in the case that bent flagella was trapped during blotting). In any case, it shows that conformational correlation between ODAs along the MTD occurs *in vivo* as well.

Structural heterogeneity of dyneins with clustering patterns allows us to propose a mechanism to generate bending motion by ODA. When arrays of dynein change their conformations and the subsequent arrays on the same MTD remain in the apo form, tension should arise and cause bending (bottom of Figure 6B) [30]. The mechanism to generate such heterogeneity is unclear but might be linked to a mechano-sensing function of dynein, which is indicated in bending of paralyzed flagella induced by mechanical stimulation [57,58] and in *in vitro* motility assay of cytoplasmic dynein [59]. This may also give a clue to a broad question—why motile cilia have dynein, complex molecules, instead of kinesin, to generate bending.

## Ciliary structure revealed by cryo-electron tomography—II. Other structures

### Radial spokes and central pair

The radial spoke (RS) is a T-shaped protein complex connecting the peripheral MTD and the central pair apparatus (CP). It is known that there are two radial spokes within one 96-nm unit in *Chlamydomonas*, while there are three radial spokes in *Tetrahymena*, sea urchin sperm flagella, and mouse respiratory cilia. Much is still unknown about the components and functions of the radial spokes. Twenty-three proteins were isolated from *Chlamydomonas* radial spokes [60], although it is not known how many copies exist in the complex. Until 2010, there were only models of the arrangement of the 23 components in the radial spoke based on chemical cross-linking and pull-down assays [61]. Diener, Rosenbaum, and their colleagues isolated L-shaped pre-assembled RS complexes with half molecular weight (12S; 710kD) and 11 components in the cytoplasm. This implies that these components are pre-assembled in the cytoplasm, transferred to the axoneme by IFT, and are finally assembled into a T-shaped RS together (23S) with the other radial spoke proteins (RSPs) [62]. According to this model, there should be at least two copies of these RSPs in the final RS assembly.

**Figure 7:**
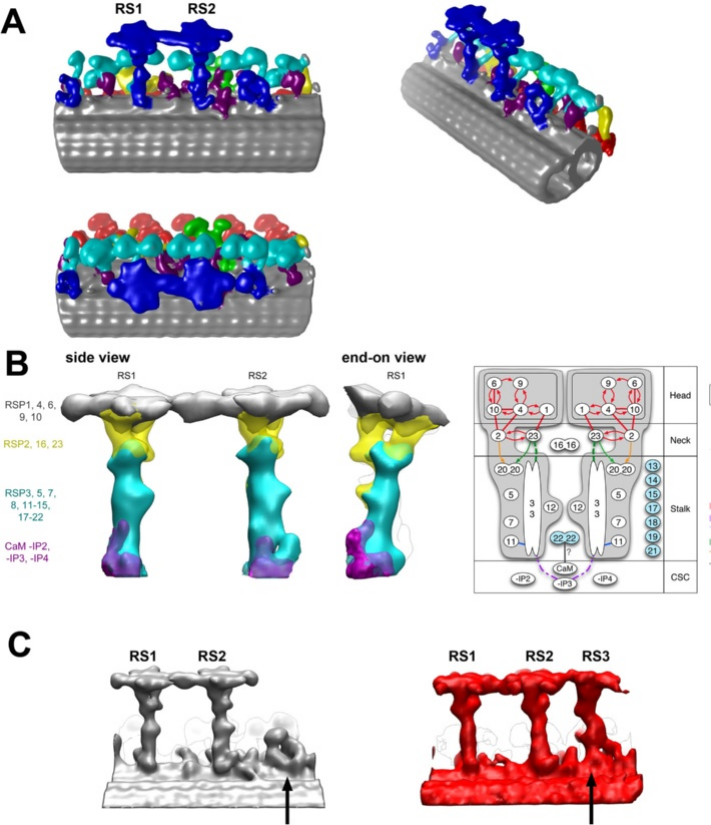
**Structure of radial spokes (RS). (A)** Various views of RS (blue) to highlight pseudo twofold symmetry of RS1 and RS2. Red: ODA. Cyan: IDA. Green: DRC. Yellow: IC/LC. Grey: MTD. In the left two panels, the proximal end is left. **(B)** Juxtaposed flagella structures of the wild-type and mutant *Chlamydomonas* lacking RSPs and the diagram of RSPs based on the structural and biochemical analyses. Modified from [66]. **(C)** RS from *Chlamydomonas* and *Tetrahymena* [66], highlighting the difference between RS1/2 and RS3 as well as the similarity between the *Tetrahymena* RS3 and the short protrusion of *Chlamydomonas* flagella (arrows).

In our tomographic reconstruction of *Chlamydomonas* flagella, the two radial spokes are similar to each other (Figure 7A), suggesting almost identical components. The entire RS structure can be divided into three parts: a head, a neck, and a stalk. We reconstructed 3D structures of flagella from the wild-type (WT) *Chlamydomonas* as well as three mutants lacking RSPs in order to locate RSPs within the structure. Both the radial spokes (Figure 7A) show nearly twofold symmetric morphology. Pseudo twofold symmetry could be explained by Diener’s model in which two pre-assembled RSPs are finally assembled into the RS. Our evaluation of the volume from the reconstruction, in which we set a threshold level to cover expected volume of tubulins in MTD, also supports the idea that there are two copies of each component in one RS. Although the reconstruction of Nicastro’s group [63,64] looks different from ours, different threshold settings can explain the discrepancy.

Location of 23 RSPs is still ongoing. Our group located RSPs 1, 4, 6, 9, and 10 in the RS head, while RSPs 2, 16, and 23 are in the RS neck. RSPs 3, 5, 7, 8, 11–15, and 17–22 are in the stalk [65,66] (left of Figure 7B). This information is based on structural analysis of mutants. Combination of BCCP-tagged protein expression and cryo-electron tomography revealed further detailed positioning and orientation of RSPs 3, 4, 6, and 11 [67]. Besides the 23 RSPs, a calmodulin binding protein complex (CSC) was identified as an essential factor for RS binding on MTD [68,69]. The current most likely model is shown in the right panel of Figure 7B, taking structural, chemical cross-linking [61], pull-down [70,71], co-purification [70], and gel overlay [72] experiments into account.

Comparative structural analysis between *Chlamydomonas* and *Tetrahymena* RSs (Figure 7C) gave us information about components and the evolution of RS3. RS1 and RS2 from both species are similar to each other, indicating similar components. However, the structure of *Tetrahymena* RS3 differs from RS1 and RS2 (Figure 7C). Interestingly, *Chlamydomonas*, which was known to have two RSs, contains structure, corresponding to the lower part of RS3 (Figure 7C; Additional file 1: Video). This unique structure, which we call the RS3 stump [65,66], stays in a mutant (*pf14*) in which the entire RSPs are missing. These findings imply that the components of *Tetrahymena* RS3 and the *Chlamydomonas* RS3 stump are not the 23 RSPs but other proteins, which are not yet identified [66].

Pseudo twofold symmetry of RS1 and RS2, prominent in the RS head part (Figure 7A), was unexpected, considering that the role of this region is interaction with CP. CP has obvious polarity, as shown by freeze-fracture deep-etch EM [73] and cryo-tomography [13,67,74]. How does the interaction occur between the symmetric RS head and the one-directional CP? The signal from the CP to the RS head might be simple mechanical pressure instead of specific biochemical signal transduction. This hypothesis was proposed based on geometry of CP, RS, and MTD [75-77] and the lack of signal transduction sequences [60,65,66]. The Kikkawa group presented experimental proof which supported this hypothesis [67]. They tagged proteins of various sizes to the RS head proteins and proved not only that the tagged proteins interrupt the flagellar motility, but that they also rescue the motility when CP misses the protrusion at the interface to the RS heads. This indicates that the signal transduction between CP and RS does not require interaction between specific amino acids, supporting the hypothesis of mechanical interaction.

### Nexin/DRC and other interdoublet linkers, MIPs

Here, we overview the other structures which follow 96-nm periodicity.

Adjacent microtubule doublets are linked by various linkers. Nexin/DRC exists between all the nine pairs of MTDs. Structure of DRC and assignment of its components was studied by the groups of Porter and Nicastro [78]. By combining cryo-electron tomography with proteomic and phosphoproteomic studies of DRC, the arrangement of DRC proteins was modeled [79]. Recently, the positions and orientation of BCCP-tagged DRC1, DRC2, and DRC4 proteins were directly revealed. These proteins (likely coiled-coil) extend from the MTD (C-termini) toward the adjacent B-tubule with the N-termini [80]. Structural knowledge, together with genetic and biochemical studies [81,82], will give insight into function of DRC. We found two more interdoublet linkers, which exist only specific pairs of adjacent MTDs (see the detail in Asymmetrical arrangement of inner arm dyneins and other proteins in *Chlamydomonas* flagella) (arrows in Figure 3C). An extra linker was found also in sea urchin sperm [83].

**Figure 8:**
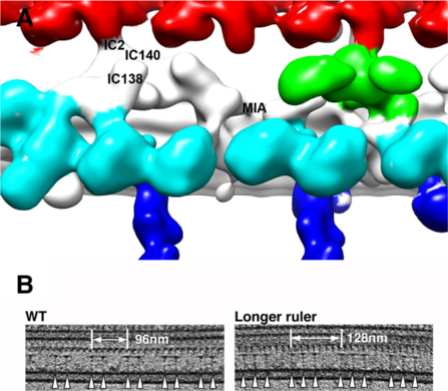
**Other structures with 96-nm periodicity. (A)** Components of IC/LC located by cryo-electron tomography based on [37-39] indicated on EMD2117 [35]. **(B)** 96-nm periodicity was elongated to 128 nm by extending coiled-coil FAP proteins [90]. RSs are indicated by triangles. Courtesy of Drs. M. Kikkawa and T. Oda.

Inside and outside MTDs, there are a number of structures connecting protofilaments [84,85]. Tektin, which was originally identified as filamentous structure inside MTD of sea urchin sperm [86], has been shown to be localized on the protofilament ribbon region [87], a finding supported by cryo-electron tomography [88]. Filamentous FAP20 was proved to be outside of MTD at the inner junction, by cryo-EM of BCCP-tagged protein, and plays an essential role for planar asymmetric motion [89]. Combination of such genetic engineering in *Chlamydomonas* and cryo-electron tomography has enormous potential. Based on the truncation and extension, coiled-coil proteins FAP59 and FAP172 are proved to be essential proteins to determine the length of the 96-nm periodic unit: by extending these proteins, they succeeded to change the periodicity to 128 nm and caused rearrangement of RS and IDA [90] (Figure 8B).

### IFT and other structures

In this section, we will review the electron tomography of ciliary structure without having 96-nm periodicity in the axoneme.

**Figure 9:**
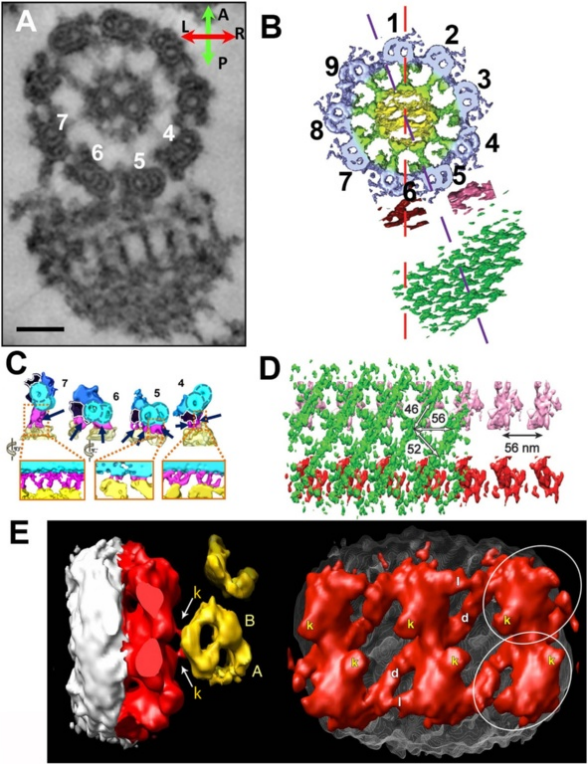
**Structure without 96-nm periodicity. (A-D)** Paraflagellar rod from *Trypanosome brucei*. **(A, B)** Cross section to visualize the axoneme (numbered) and the paraflagellar rod. The definition of the numbering MTDs is different from *Chlamydomonas*. **(C)** Interface structure between the axoneme and the paraflagellar rod. **(D)** 2D crystal structure of the paraflagellar rod seen from the side (perpendicular to the axoneme). (A, C) From [92]. **(B, D)** From [91] with permission. **(E)** IFT long train [93], which is responsible for anterograde transport. Left: view from the tip. Right: view from MTD. Structure at the interface to MTD, likely kinesin, is indicated as “k”. A- and B-tubules are also labeled. ©Pigino et al. [93]. Originally published in J. Cell Biol. doi: 10.1083/jcb.200905103

The axoneme of *Trypanosoma brucei*, which produces a bihelical motion, contains a structure called a paraflagellar rod next to the axoneme. The paraflagellar rod consists of layers of two-dimensional lattice-like protein networks. Two electron tomography works on this structure highlight compensatory aspects obtained from ice-embedded and stained specimens. Cryo-electron tomography and subtomogram averaging enabled 3D reconstruction of the unit cell of the crystalline structure [91] (Figure 9B,D). They demonstrated distortion of the unit cells, corresponding to the local curvatures of the axoneme, and proposed the mechanism of how the paraflagella regulate the waveform of *Trypanosoma* flagella (jackscrew model). Dual axis tomography of stained sample provided a direct view of the entire flagella without averaging, revealing the detailed geometry of the joint between the axoneme and the paraflagellar rod [92] (Figure 9A,C).

Intraflagellar transport (IFT) is the key complex for ciliogenesis. 3D structure of IFT long trains reconstructed by subtomogram averaging from the flat-embedded sections of *Chlamydomonas* flagella [93] showed interesting pseudo twofold symmetry, which was not expected considering the clear polarity of MTDs (Figure 9E). Two contact points between IFT and MTD, which are likely kinesins, also follow twofold symmetry, although kinesins must bind to the microtubule with polarity. We need higher resolution to reveal how this pseudo twofold structure interacts with MTD. The technical difficulty is that only one or two IFTs are found in one tomogram of flagella. A recently established *in vitro* purification technique of IFT [94] could enable single particle analysis or tomography with high efficiency. Hopefully, EM structure of IFT trains will be fitted to atomic structures of components [95] in the future.

The flagellar tip, which is the unloading dock of IFT, should be the next interesting target of structural analysis. The central microtubule cap and the distal filament were reported as structure found by negative stain EM at the tip of CP and MTD, respectively [96-98]. The first work of electron tomography of flagellar tips from *Chlamydomonas* and *Trypanosoma* prepared by freeze substitution and staining describes the arrangement of microtubules and densities from unidentified proteins [22]. However, 3D analysis to address molecular arrangement is still missing. Recently, the CEP104/FAP256 protein was located at the tip [99]. Further structural analysis of the tip complex is awaited.

### Basal body

**Figure 10:**
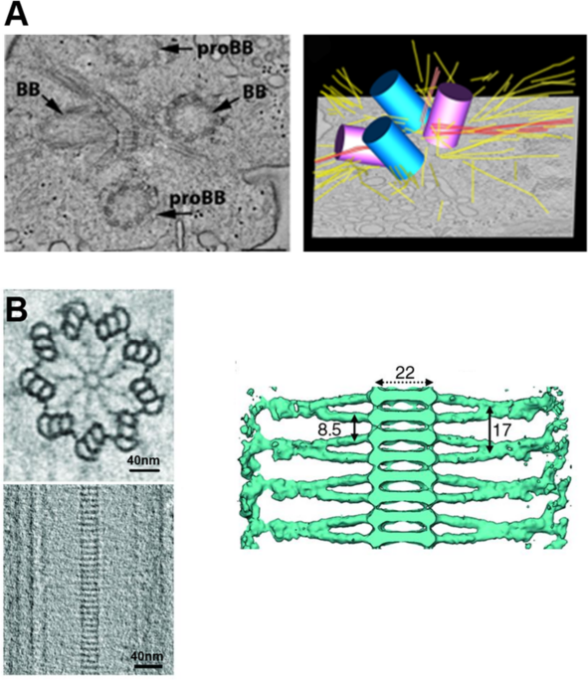
**Structure of basal bodies revealed by electron tomography. (A)** Room temperature tomography visualized microtubule networks around basal bodies. From [101] with permission from Wiley. **(B)** Reconstruction from cryo-electron tomography indicates the hook structure on the microtubule triplet (top left), stacked cartwheels (bottom left and right), and branched spokes (right). From [105]. Reprinted with permission from AAAS. *BB* basal body. *proBB* pro-basal body.

Structural analysis of basal bodies by electron tomography was initiated by O’Toole, Dutcher, and their colleagues by tomography using sections from *Chlamydomonas* cells prepared by high pressure freezing and freeze substitution [100]. In addition to high contrast, which enables direct observation of features without averaging, serial sectioning allows 3D visualization of thick sections (600 nm in the case of a basal body) by serial tomography. Another advantage of tomography at room temperature is double-axis tilting without a highest end microscope. With this method, features including a cartwheel, transitional fibers, and rootlet MTs were directly visualized from a basal body [100]. They applied the same technique to visualize microtubule organization during duplication and elongation of the basal bodies from mitotic *Chlamydomonas* cells [101] (Figure 10A). The way of bidirectional elongation of B- and C-tubules along the A-tubule is similar to that in the human centriole, described by cryo-electron tomography [102].

There are only a few structural works of the basal body using cryo-electron tomography and subtomogram averaging, due to the difficulty to improve signal-to-noise ratio in the absence of 96-nm periodicity. Nevertheless, ninefold averaging still improves signal-to-noise ratio. The cartwheel, which is considered to be essential for the ciliogenesis from nine microtubules [103,104], has ninefold symmetry and thus gets benefit from subtomogram averaging. The cartwheel ring structure was well resolved [105,106] (Figure 10B), using exceptionally long stacks of cartwheels in *Trichonympha*. The structure averaged from subtomograms and ninefold symmetrized fitted well to the atomic models of nine SAS-6 proteins forming a ring. The spoke connecting cartwheel rings and the microtubule triplet was proved to be in a branched structure (right of Figure 10B). The interface between the cartwheel spoke and the microtubule triplet is characterized by a unique “hook” structure, which was also shown by averaged structure of the basal body triplet [107].

## Outlook and future development

### Limitation of cryo-ET

The current best resolution of cryo-electron tomography is about 25 Å. This is mainly limited by radiation damage. Can it be improved? The recent progress of direct electron detectors, which replaced negatives and classical digital cameras, enabled single particle analysis to reach nearly atomic resolution [25]. Direct detectors have improved S/N at the high resolution range and thus allow us to obtain more signals at high resolution and more precise alignment. In the case of tomography, the resolution is limited by radiation damage due to multiple exposures. Therefore, such drastic improvement of resolution as seen in single particle analysis is not expected in tomography by using direct detectors. However, direct detectors might allow subtomogram alignment at much lower exposure and thus subtomogram gives averaging with less radiation damage, which in turn results in higher resolution. Due to poor contrast of cryo-electron micrographs, we always need to average many subtomograms to extract information. Development of the phase plate [108] may help this situation and enable us to obtain structural information at the comparative resolution from fewer averages in the future.

Whatever advantage we pursue in the near future, resolution of cryo-electron tomography (including subtomogram averaging) will not reach atomic resolution. In the case of cilia, consisting of >600 proteins [109], it is not possible to identify them based on the structure directly. The list of proteins obtained by proteomics and 3D structure by tomography at 25 Å must be linked. In our previous works, we compared mutants lacking dynein and radial spoke proteins to locate them in tomograms [34,35]. To apply this approach, we generally need to systematically make deletion mutants. Another approach is labeling, either chemical labeling or genetic tagging. Recently, the Kikkawa group succeeded in combining expression of genetically tagged radial spoke proteins with *Chlamydomonas* deletion mutants of these radial spoke proteins and dynein f intermediate chains to locate the N- and C-termini of these proteins [67]. To facilitate this approach, we would like to carry out systematic tagging. We should either mutate wild-type genes to tagged genes or express tagged genes in cells with the wild-type gene knocked out/down. Whereas mutation based on homologous recombination is not established in *Chlamydomonas*, successful knockdown by amiRNA has been reported [68]. RNAi is applied to engineer cilia from planaria as well [110]. However, there is no report of mutant expression in knocked down cells. Expression of tagged protein in knockout mice might be an option. There has been no report of locating proteins in cilia by specific antibody labeling. The complex structure of the axoneme likely inhibits antibodies (even Fab) from binding epitopes. Smaller artificial labels such as DARPins [111] may open the possibility of specific labeling.

Cryo-tomography has been contributing to cilia research utilizing the 96-nm periodicity of the axoneme. Analysis of the other components, which do not follow the periodicity, is relatively behind. Heterogeneity must be dealt with. Our group has already revealed heterogeneity along individual microtubule doublets and among nine doublets [35,41]. Similar heterogeneity exists along doublets of human cilia in the outer dynein components [112]. Heterogeneity must be examined among cilia from the same ciliated tissue. For this purpose, image classification techniques and correlative electron/optical microscopy must be combined.

### Combination with other methods

To study molecules which, unlike dynein and radial spokes, do not form 96-nm periodicity along the entire length of the axoneme, we need to locate them. In our work, we distinguished the proximal region by selectively averaging <2 μm from the basal body. We could deal with more complex localization, if it exists, if we have prior knowledge about localization of the molecule within the cilia. Correlative light/electron microscopy is a technique to compare images obtained from light and electron microscopy. It enables us to locate proteins with fluorescent probes by fluorescent microscopy and reconstruct high resolution 3D structure from EM. In cryo-correlative microscopy, frozen grids are observed in a specially designed cryo-stage installed in the optical microscope to record coordinates of the objects of interest and then transferred to cryo-EM (review in [113]). This technique is used to study localization of target molecules in the cell [114]. The challenge is to detect fewer probes on frozen grids. The long distance between the object lens and cryo-specimen limits resolution and sensitivity.

### High-throughput for diagnosis

Can we use cryo-ET of cilia as a tool to diagnose ciliopathies? The answer would be Yes if high throughput data collection and analysis will be possible in the future. Currently, ultrastructural observation to diagnose ciliopathies is mainly carried out by EM of plastic-embedded sections and has identified cilia lacking dynein arms [115,116], radial spoke proteins [117,118], and DRC [82]. Deletion of other components might be too subtle for direct visualization of chemically fixed cilia. If cryo-ET data acquisition and averaging of 96-nm periodic units are fully automated, it would be possible for non-cryo-ET experts to reconstruct 3D structure and diagnose ciliopathy based on high resolution 3D structure. Data acquisition has potential for automation. Microscopes are available with a stable stage and semi-automatic tomography acquisition, in which the operator indicates where on the grid axonemes are located at low resolution and a program collects tomographic datasets at these locations. Data analysis should be modified to be more user-friendly for this purpose.

## Conclusion

3D structural analysis from cryo-electron tomography has given insight into cilia research from the scale of molecules to the scale of organelles. We located dynein isoforms in *Chlamydomonas* flagella and positioned radial spoke proteins. 3D image classification proved nucleotide-induced conformational change of dyneins and interesting distributions of multiple forms of dynein in the presence of nucleotides in cilia. Discussion at near atomic resolution is possible by fitting atomic models to tomograms. It also should have a potential to expand to contribute to cilia research at the tissue level, by combining with other specimen preparation methods. After more than 10 years since this method was applied to cilia, we are now in the next phase of research.

## Additional file

Additional file 1:
**Video to show the assignment of the main components of cilia based on cryo-electron tomography.**

## Abbreviations

BCCP: biotin carboxyl carrier protein
CP: central pair apparatus
CSC: calmodulin binding protein complex
DHC: dynein heavy chain
DRC: dynein regulatory chain
ET: electron tomography
FEG: field emission gun
IC/LC: intermediate and light chains
IDA: inner dynein arm
IDL: inter-doublet linker
IFT: intraflagellar transport
MTD: microtubule doublet
ODA: outer dynein arm
RS: radial spoke
RSP: radial spoke protein
S/N: signal-to-noise ratio
WT: wild type
